# A roadmap of strain in doped anatase TiO_**2**_

**DOI:** 10.1038/s41598-018-30747-5

**Published:** 2018-08-24

**Authors:** N. Kelaidis, A. Kordatos, S.-R. G. Christopoulos, A. Chroneos

**Affiliations:** 1 Faculty of Engineering, Environment and Computing, Coventry University, Priory Street, Coventry, CV1 5FB United Kingdom; 2 Department of Materials, Imperial College, London, SW7 2AZ United Kingdom

## Abstract

Anatase titanium oxide is important for its high chemical stability and photocatalytic properties, however, the latter are plagued by its large band gap that limits its activity to only a small percentage of the solar spectrum. In that respect, straining the material can reduce its band gap increasing the photocatalytic activity of titanium oxide. We apply density functional theory with the introduction of the Hubbard + U model, to investigate the impact of stress on the electronic structure of anatase in conjunction with defect engineering by intrinsic defects (oxygen/titanium vacancies and interstitials), metallic dopants (iron, chromium) and non-metallic dopants (carbon, nitrogen). Here we show that both biaxial and uniaxial strain can reduce the band gap of undoped anatase with the use of biaxial strain being marginally more beneficial reducing the band gap up to 2.96 eV at a tensile stress of 8 GPa. Biaxial tensile stress in parallel with doping results in reduction of the band gap but also in the introduction of states deep inside the band gap mainly for interstitially doped anatase. Dopants in substitutional positions show reduced deep level traps. Chromium-doped anatase at a tensile stress of 8 GPa shows the most significant reduction of the band gap as the band gap reaches 2.4 eV.

## Introduction

Fujishima and Honda^1^, essentially attracted the interest of the scientific community to titanium oxide (TiO_2_) for photocatalysis applications. Thereafter, TiO_2_ and other metal oxides have been intensively investigated because of their catalytic activity, long lifetime of photon generated carriers, and high chemical stability^2–10^. Among the polymorphs of TiO_2_, anatase has the highest photocatalytic activity but it is plagued by its large band gap (3.2 eV). This limits the activity to the ultraviolet range i.e. ~5% of the solar spectrum^7^. Ideally, for photocatalysis the band gap should be close to 2 eV with the positions of the band edges being compatible with the redox potential of water^11^.

Introducing non-metallic dopants such as nitrogen (N) or carbon (C), metallic dopants such as chromium (Cr) and iron (Fe), or intrinsic defects (oxygen or titanium vacancies and interstitials) in the TiO_2_ lattice has been proposed as a way to modify the band structure in order to reduce the band gap thus enhance the visible light response^3,12–14^. Apart from doping, mechanical stress is another efficient way to design the electronic and defect processes in metal oxides^15–18^. It is established that volume change via an applied force can lead to changes in the band gap and electronic structure of semiconductors, effectively modifying their electrical properties^19–24^.

Epitaxial growth of anatase can be a potential method for the introduction of stress. Due to the lattice mismatch between adjacent layers, when a thin film is grown epitaxially stress is introduced to the top layer. In order to improve its photocatalytic properties, anatase has been grown epitaxially on materials with similar lattice constant such as LaAlO_3_ (LAO) and SrTiO_3_ (STO) perovskite substrates, on rutile by DC magnetron sputtering, on SnO_2_, or on double heterostructures like TiO_2_/ZnO/TiO_2_^25–28^. To assist in the interpretation of experimental data, but mainly to propose a pathway for improved photocatalytic performance, the effect of strain along with the incorporation of defects needs to be examined. In the present study we examine the combined effect of strain along with the incorporation of defects for the anatase TiO_2_. We use density functional theory (DFT) calculations to investigate the impact of compressive and tensile stress up to 8 GPa on the electronic structure of undoped and doped-TiO_2_. Initially, we examine the effect of the type of stress, i.e. hydrostatic, biaxial and uniaxial, on the electronic properties of anatase. Then we concentrate on biaxial stress, as the most technologically relevant and we examine the combined effect of stress and band gap engineering methods such as (a) introduction of defects (oxygen or titanium vacancies and interstitials), (b) metallic dopants (Fe, Cr) and (c) non metallic dopants (N, C) as interstitials or substitutional atoms.

## Results and Discussion

### Impact of stress on perfect anatase

Here we considered the anatase (tetragonal with space groups I4/amd) polymorph of TiO_2_ as it has superior photocatalytic properties compared to the rutile and brookite polymorphs^29–33^. The calculated lattice parameters of anatase, a = 3.806 Å and c = 9.724 Å, are in excellent agreement with previous experimental and theoretical results^31–34^.

**Figure 1:**
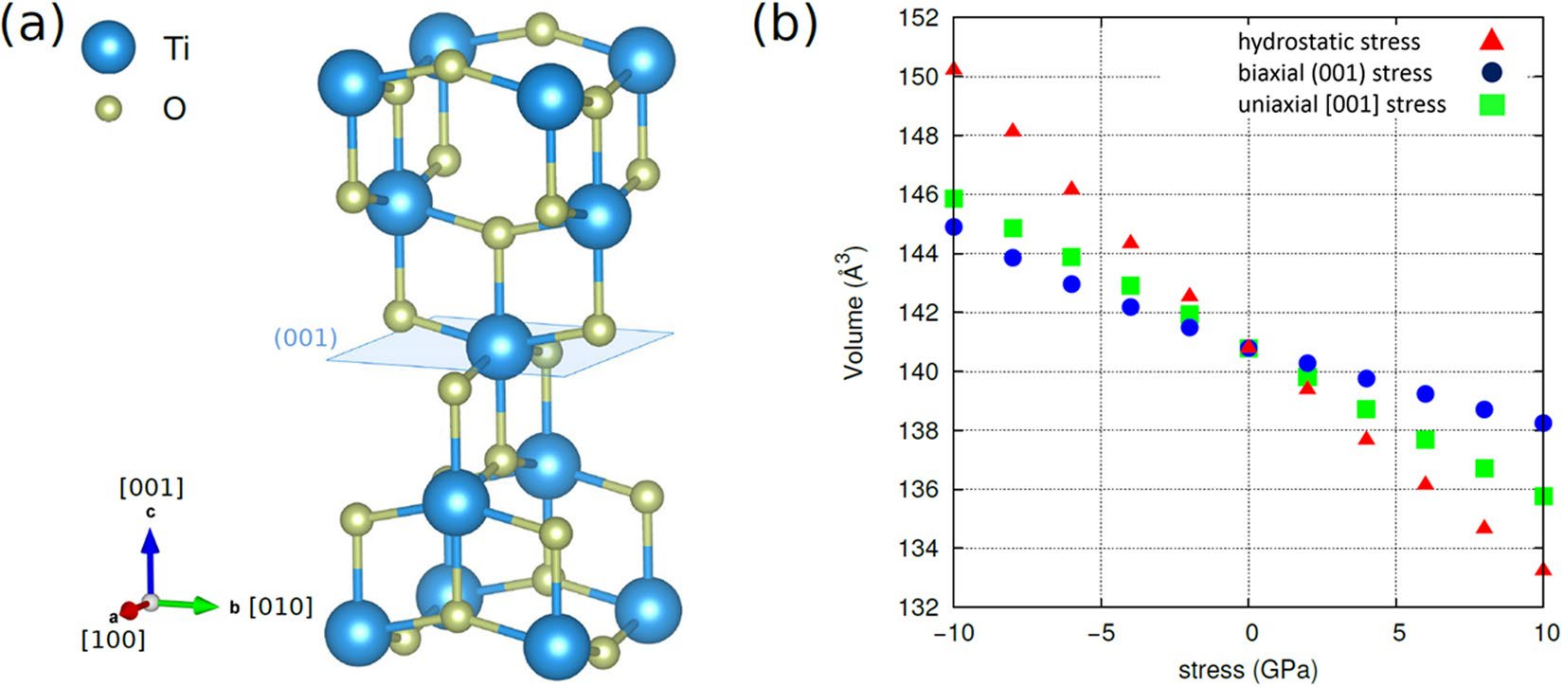
(**a**) Anatase conventional cell (**b**) volume of primitive cell with respect to stress.

We have performed a series of calculations under fixed external stress (ranging from 0 to 10 GPA for the perfect structure) for both compressive and tensile stresses. For completeness, the effect of hydrostatic, biaxial and uniaxial stress was examined although some of these stress conditions will be very difficult to attain experimentally. In Fig. 1(a) the unit cell of anatase is shown. Biaxial stress is applied to the (001) plane (defined by the x,y axes in Fig. 1). The effect of biaxial stress on the (100) plane (defined by y,z axes in Fig. 1) has also been examined but it is not presented here as it leads to a smaller decrease of the band gap (refer to the Supplementary Information, Table S1).

Uniaxial stress was applied along directions [100] and [001] (a and c axis in Fig. 1a). The effect of strain on total volume and band gap is more pronounced on the [001] axis (refer to Supplementary Information, Table S1), in agreement with previous studies, where it is considered as a soft axis due to its higher compressibility^20^.

The effect of stress on the total unit cell volume is shown in Fig. 1(b). As expected, hydrostatic stress has the maximum effect on total volume. Conversely, biaxial stress on the (001) plane results in less volume change than when exerting uniaxial stress along the [001] axis. As pointed out, this is due to the different compressibility of the axes and the different elastic constants of anatase (c_11_ = c_22_ and c_33_).

**Figure 2:**
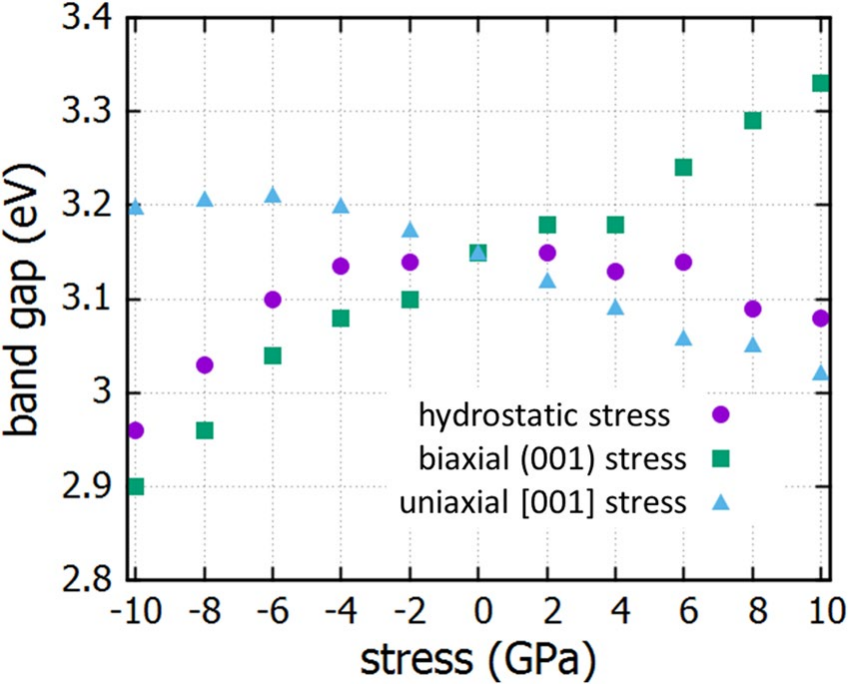
Band gap with respect to stress for undoped anatase for hydrostatic, biaxial and uniaxial stress.

We performed electronic structure calculations and derived the band gap for each case of anatase under stress. The Hubbard + U model was applied in our calculations, for a correct estimation of the band gap, in order to address the correlation effects of localized electrons onsite Coulomb repulsions. U is set at 8.2 eV for the Ti 3d orbitals, in accordance with previous work^14^. The effect of stress on the band gap of TiO_2_ for uniaxial, biaxial and hydrostatic stress is shown in Fig. 2. We observe that the minimum band gap for anatase can be obtained for the case of biaxial tensile stress, reaching 2.96 eV (closer to the optimal gap of 2.0 eV) for the tensile stress of 8 GPa. The band gap as a function of stress is not linear and differs from a previous study (although similar trends are observed) where the PW91 generalized gradient approximation GGA was applied, without the use of Hubbard + U correction^20^. The band gap versus biaxial stress when fitted with a second order polynomial, is approximately:

$$B.G.\,(eV)=3.142\,eV+19.8\frac{meV}{GPa}P-0.265\frac{meV}{{(GPa)}^{2}}{P}^{2}$$

Uniaxial compressive stress is also an effective method to reduce the band gap, as we observe in Fig. 2. Compression in the z axis yields expansion in the xy plane, therefore the case of uniaxial stress along the [001] axis and biaxial stress in the (001) plane are actually similar. Biaxial stress can be achieved by suitable growth methods and is a technologically interesting approach in order to introduce stress to a substrate. We will therefore focus more on biaxial stress in what follows.

### Impact of strain on intrinsic defects

We examine first the effect of strain on intrinsically defective anatase. Introducing oxygen vacancies is known to improve the anatase photocatalytic properties^35^. Anatase can be easily grown with oxygen deficiency as under normal growth conditions it is substoichiometric with an excess of titanium. The titanium interstitials or oxygen vacancies (or both) are responsible for an *n*-type character. Alternatively, growth can be achieved with the introduction of titanium vacancies (V_Ti_) in the crystal of anatase which then shows *p*-type conductivity and again improved photocatalytic performance^36^.

**Figure 3:**
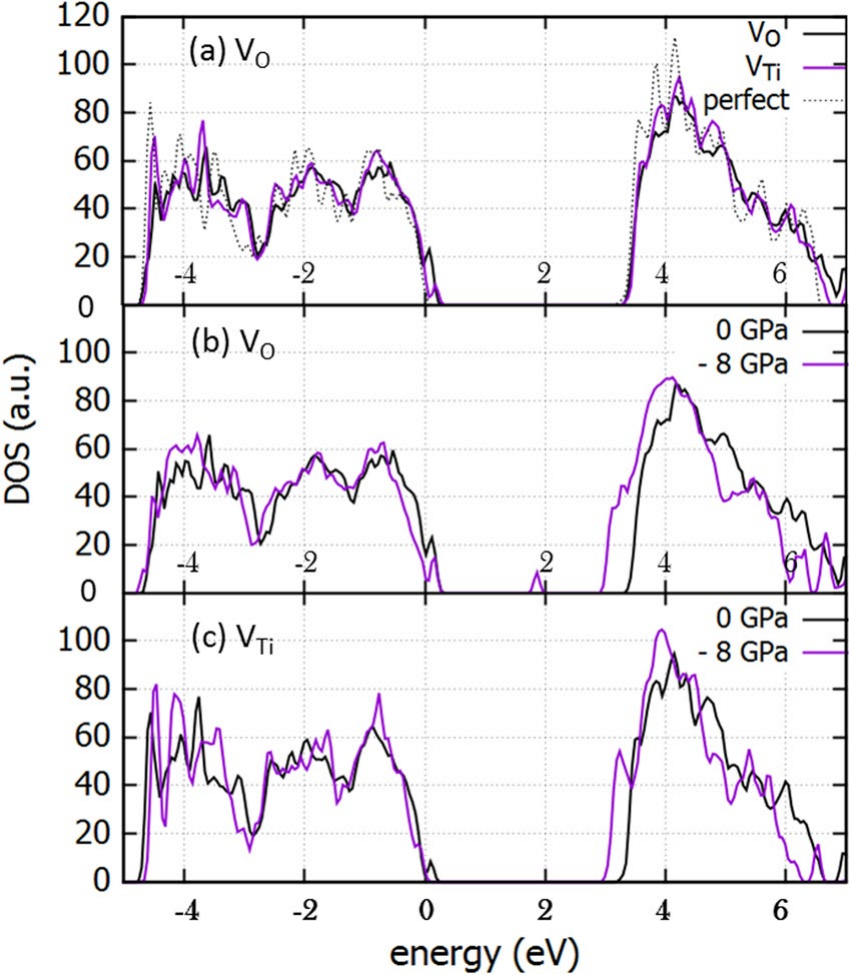
Densities of states for unstressed and biaxial (001) tensile stressed (–8 GPa) anatase: (**a**) Perfect supercell (**b**) defective supercell with an oxygen vacancy V_O_ (**c**) defective supercell with a Titanium vacancy V_Ti_.

We calculated the effect of stress on the electronic properties of anatase when intrinsic dopants are present, starting with a perfect 3 × 3 × 1 supercell of anatase TiO_2_ (which consists of 36 Ti and 72 O atoms) and introducing a vacancy of oxygen or titanium. This amounts to an approximate 2.8% concentration of dopants. In Fig. 3(a), the effect of oxygen and titanium vacancies on the density of states on an anatase supercell is shown, in comparison with the perfect cell of anatase. The introduction of an oxygen vacancy in the supercell, according to our calculations leads to an increased density of states near the valence band (VB) edge, leading to a reduction of the band gap of 0.08 eV whereas in the case of a titanium vacancy, a tail formed in the subgap region reduces the band gap by 0.12 eV (refer to Fig. 3a). When introducing biaxial tensile stress the band gap is further reduced. In Fig. 3, the effect of biaxial tensile stress of 8 GPa on the electronic properties of anatase supercell with oxygen vacancies (refer to Fig. 3b) and with titanium vacancies is shown (refer to Fig. 3c). The reduction of the band gap with the biaxial tensile stress is significant, about 0.43 eV and 0.28 eV, reaching 2.67 eV and 2.90 eV for oxygen and titanium vacancies respectively.

### Impact of strain on doped anatase: metallic dopants

A range of metallic dopants has been used to enhance the photocatalytic properties of TiO_2_, e.g. Cr, Fe, Mo, Ni amongst others^37^. In this study we examine the effect of strain on the electronic properties of anatase doped with Cr, Fe as interstitials and substitutional metallic dopants.

**Figure 4:**
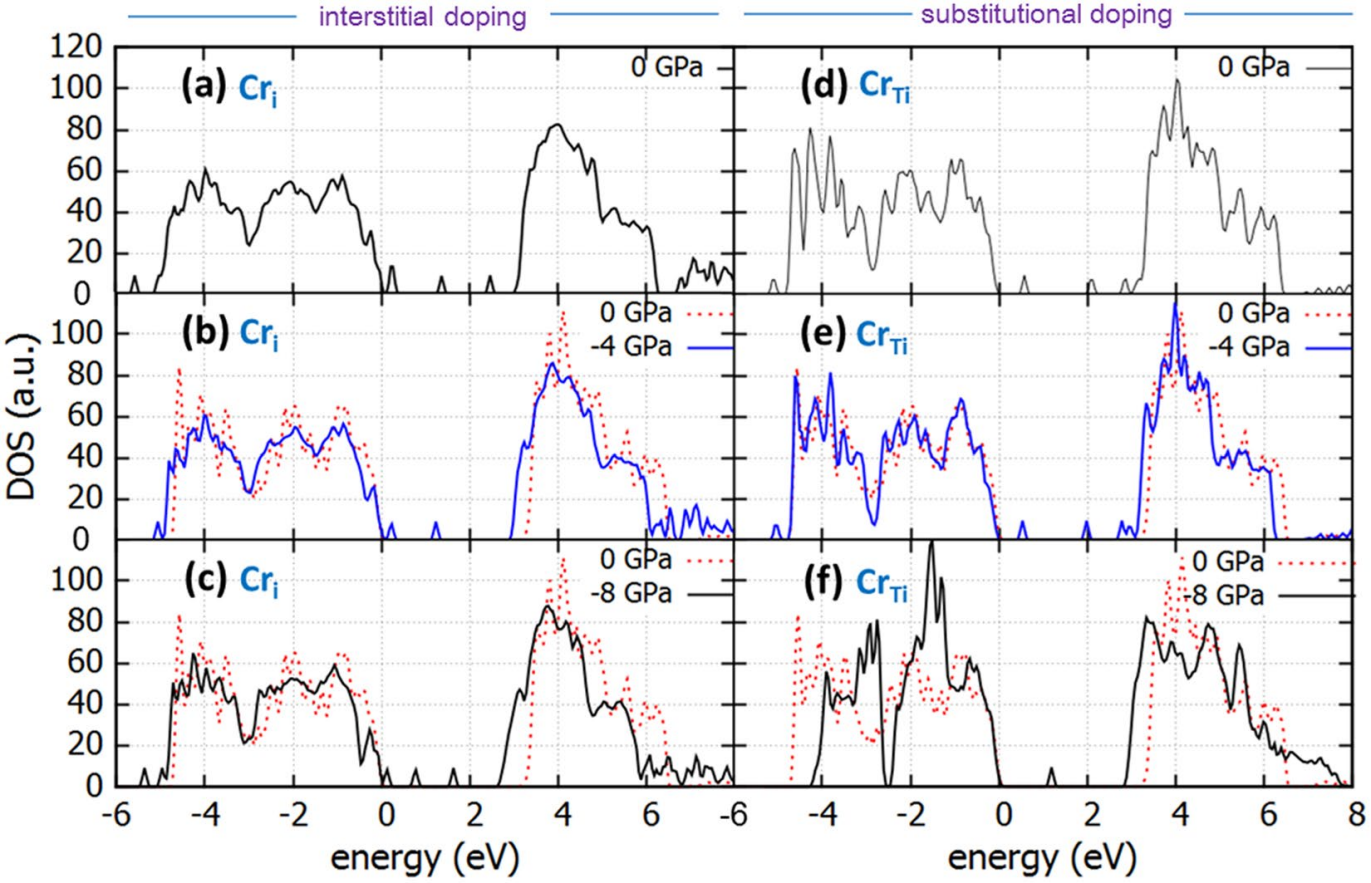
DOS of Cr-doped TiO_2_ with Cr as interstitial dopant (Cr_i_) under biaxial tensile stress of (**a**) *0 GPa* (**b**) *4 GPa* (**c**) *8 GPa* and DOS of TiO_2_ with Cr as substitutional dopant (Cr_Ti_) under biaxial tensile stress of (**d**) *0 GPa* (**e**) *4 GPa* and (**f**) *8 GPa*. Dotted line is the DOS of the perfect unstrained structure.

**Figure 5:**
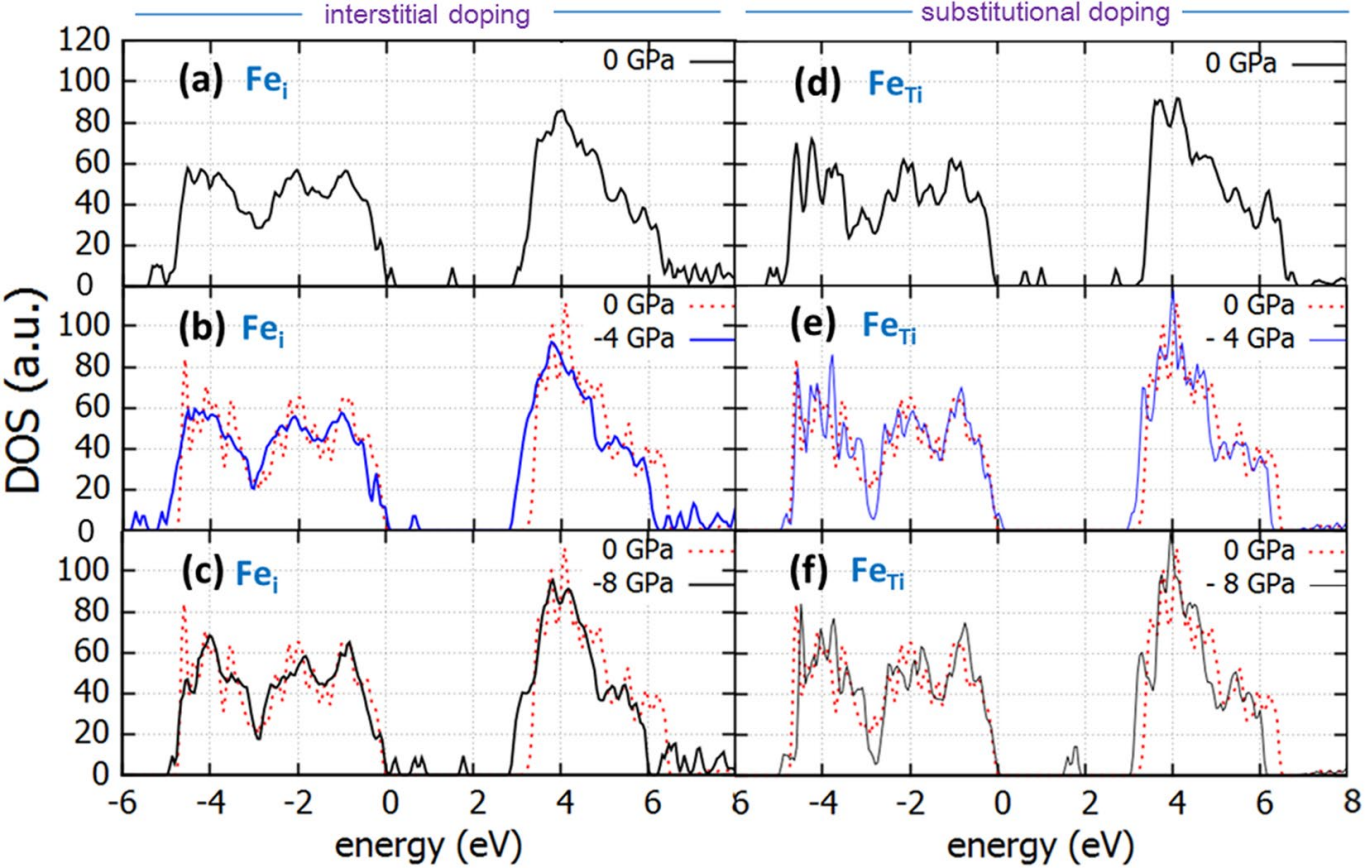
DOS of Fe-doped TiO_2_ with Fe as interstitial dopant (Fe_i_) under biaxial tensile stress of (**a**) *0 GPa* (**b**) *4 GPa* (**c**) *8 GPa* and DOS of TiO_2_ with Fe as substitutional dopant (Fe_Ti_) under biaxial tensile stress of (**d**) *0 GPa* (**e**) *4 GPa* and (**f**) *8 GPa*. Dotted line is the DOS of the perfect unstrained structure.

The density of states for interstitially and substitutionally doped anatase is shown for the stresses of 0 GPa, −4 GPa and −8 GPa for anatase doped with Cr in Fig. 4 and with Fe in Fig. 5 (shown in comparison with the perfect un-stressed anatase for reference). Here we took into consideration the strong Coulomb interactions for the localized d electrons of Fe and Cr as well, therefore introducing the *U* term parameter for the 3d states of Cr and the 3d states of Fe. The choice for the Hubbard term in this work was 4 eV for Cr and 6.4 for Fe, in accordance with previous work^38,39^.

For interstitially Cr-doped anatase, the addition of Cr reduces the band gap for the unstressed cell and is further reduced with tensile stress. A presence of additional energy levels at the onset of valence band (refer to Fig. 4a–c) is important. These states are present only when the calculations are performed with the addition of the *U* term for Cr, therefore are absent when only the *U* term for Ti is taken into account. Hence, taking this band tail into account, the presence of Cr interstitials reduces the band gap of unstressed anatase to 2.64 eV and to 2.43 eV for tensile stress of 8 GPa (please refer to Table 1). If the *U* term for Cr is not taken into account the band tail is not present and these numbers are 2.99 eV and 2.6 eV for the unstressed and the 8 GPa tensile stress respectively. The significant modification of the band gap comes also with the inevitable presence of midgap states (refer to Fig. 4). A deep level is present at 1.4 eV for the unstressed structure and shifts to approx. 1.2 eV for 4 GPa and to 0.8 eV for 8 GPa.

**Table 1:** Band gap (eV) of TiO_2_ anatase under tensile stress for Cr and Fe -doped anatase in interstitial or substitutional position.

Tensile Stress (GPa)	BG (eV) Cr_i_	BG (eV) Cr_Ti_	BG (eV) Fe_I_	BG (eV) Fe_Ti_
0	2.64	2.75	2.70	3.22
4	2.58	2.70	2.75	2.83
8	2.43	2.79	2.51	2.98

When Cr is substitutional to Ti, the band gap is significantly reduced to 2.8 eV for the unstressed structure (refer to Fig. 4). This is mainly due to states that appear near the conduction band (CB) and act as a band tail. The effect of tensile stress is not as beneficial as in the case of interstitial doping initially, as the band gap is slightly reduced for the tensile biaxial stress of 4 GPa to 2.7 eV. However, further increase of tensile stress to 8 GPa reduces the band gap. With the aid of the band tail near the CB, the band gap is reduced to 2.28 eV, which is the minimum in this work. Also, an additional mid gap state is present for all stresses, which appears at 0.8 eV to 1.2 eV above the VB maximum, depending on stress. Therefore, doping anatase with Cr is beneficial, with tensile stress adding up to the band gap modification and further reduction of the band gap for both interstitial and substitutional doping.

When introducing Fe as interstitial dopants in the structure, the band gap is affected in a mode similar to Cr (refer to Fig. 5). For interstitial doping, the introduction of Fe significantly reduces the band gap (to 2.7 eV), whereas the tensile stress proves beneficial further reducing it to 2.5 eV (Table 1). However, doping with Fe in a Ti substitutional position does not reduce the band gap in our calculations and the application of biaxial tensile stress has a very limited effect, therefore rendering this method ineffective (Table 1).

In experimental conditions, but interstitial and substitutional dopant atoms can be present. According to the above results, Cr should be preferred than Fe for which anatase should be grown stoichiometric in order for the Fe dopants to obtain interstitial positions.

### Impact of strain on interstitially defected anatase: non-metallic dopants

Carbon or nitrogen doping of anatase has been investigated (both experimentally and theoretically) as an effective method to increase the photocatalytic properties of anatase. As expected, substitutional carbon positions are favoured when there is an oxygen deficiency during growth whereas substitutional positions are more likely when there are O-rich conditions^40^. Similar conditions apply for nitrogen^41^.

**Figure 6:**
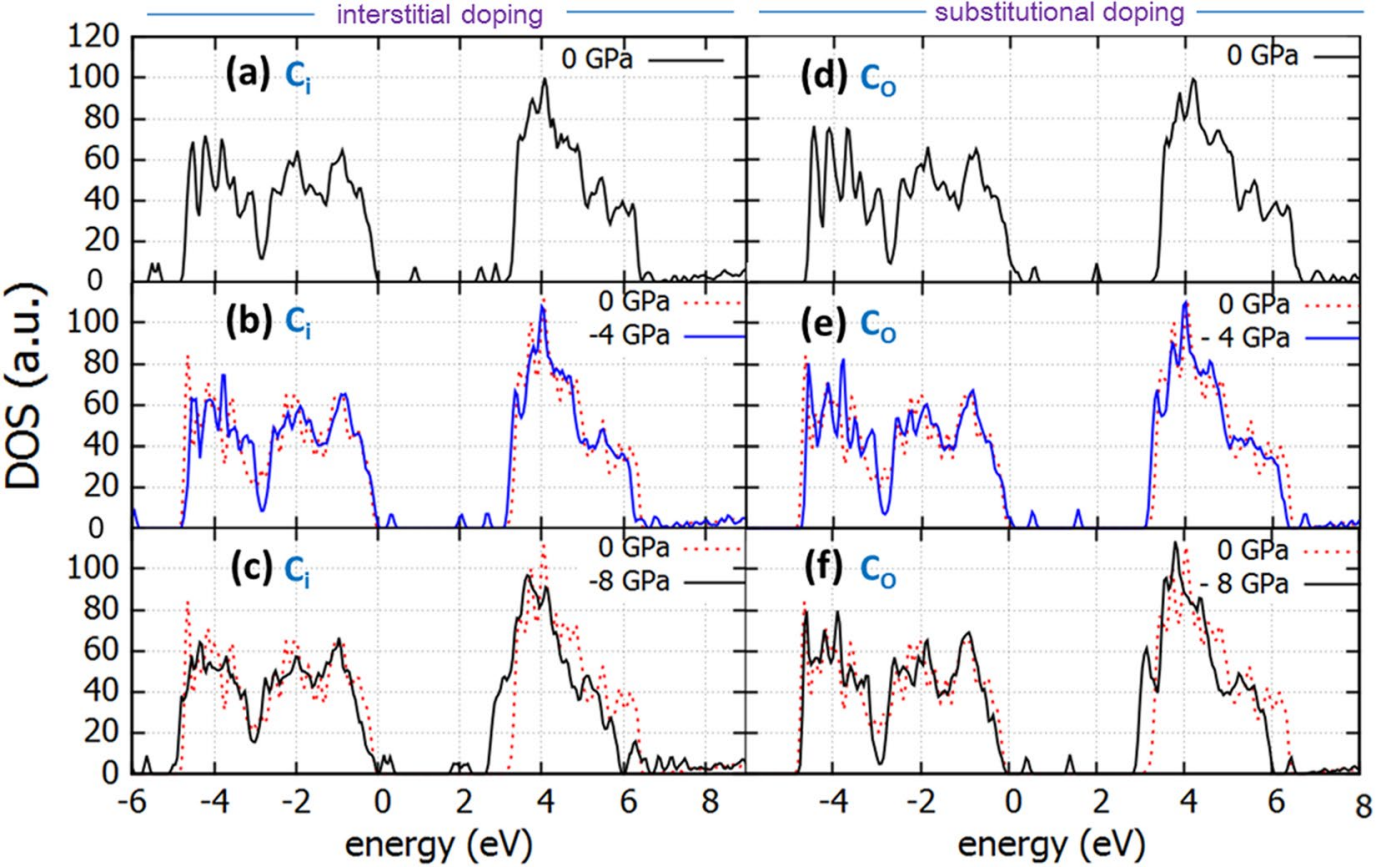
DOS of C-doped TiO_2_ with C as interstitial dopant (C_i_) under biaxial tensile stress of (**a**) *0 GPa* (**b**) *4 GPa* (**c**) *8 GPa* and DOS of TiO_2_ with C as substitutional dopant (C_O_) under biaxial tensile stress of (**d**) *0 GPa* (**e**) *4 GPa* and (**f**) *8 GPa*. Dotted line is the DOS of the perfect unstrained structure.

We have examined the effect of C and N as interstitials (C_i_ or N_i_) or substitutional in oxygen positions (C_O_ or N_O_). In Fig. 6 the effect of tensile biaxial stress on the density of states of carbon doped anatase is shown for the stresses of 0 GPa, 4 GPa and 8 GPa for C_i_ (Fig. 6a–c respectively) and C_O_ (Fig. 6d–f). Computations show that tensile stress is beneficial especially for the case of C_i_ (interstitially doped anatase) as it reduces its band gap from 3.13 eV (0 GPa) to 2.67 eV (8 GPa). This is lesser so for C_O_ which appears with a smaller band gap for unstrained structure (3.00 eV, due to additional band tail at the CB edge) but reduces only to 2.93 eV for –8 GPa. Additional density of states are present in the band gap in both cases. In the presence of C_i_, impurity levels are present at 0.9 eV, 2.5 eV and 2.9 eV above the VB edge which shift to smaller energies with strain. These are attributed to C 2p localised states^42^. For higher tensile strain (refer to Fig. 6c), these states are situated very close to the VB edge, therefore are more likely to contribute to photoactivity. For C_O_ species, deep level states are situated at 0.5 eV and 2 eV above the VB edge (refer to Fig. 6d).

**Figure 7:**
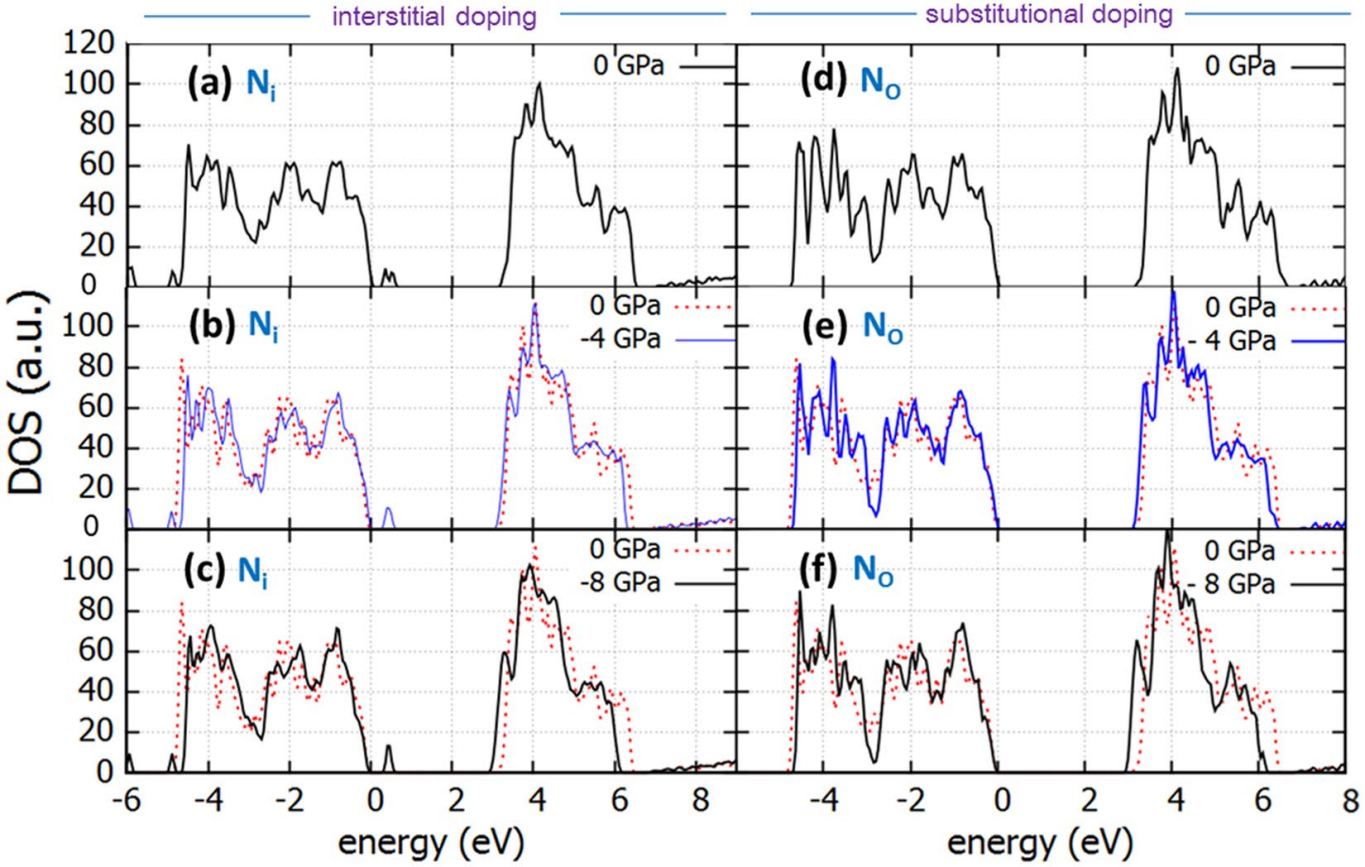
DOS of N-doped TiO_2_ with N as interstitial dopant (N_i_) under biaxial tensile stress of (**a**) *0 GPa* (**b**) *4 GPa* (**c**) *8 GPa* and DOS of TiO_2_ with N as substitutional dopant (N_O_) under biaxial tensile stress of (**d**) *0 GPa* (**e**) *4 GPa* and (**f**) *8 GPa*. Dotted line is the DOS of the perfect unstrained structure.

When introducing N in the anatase supercell either as interstitial atom or oxygen substitutional (refer to Fig. 7), the anatase band gap is reduced with biaxial tensile stress in a similar way as in the undoped case (i.e. to 3.06 eV for 4 GPa and 2.97 eV for 8 GPa). Additionally, there is a formation of a band tail near the valence band, consistently with previous results, which has been proved beneficial to the photocatalytic performance of anatase introducing the N 2p states at 0.35 eV from the VB maximum. In effect, these states effectively reduce the band gap (~2.4 eV) in agreement with experiment and in essence enhances the visible light response of anatase^43^. Notably, when N is in substitutional position, the band tails are absent from the DOS calculations.

### Summary

The present study can be useful for the interpretation of experimental results. The increase of the photocatalytic efficiency by doping may be weakened by recombination losses (i.e. interface states), whereas maintaining high strain may be a challenge. Here we considered doping of about 2.8% for various dopants (Cr, Fe, C and N). Doping in conjunction with biaxial tensile stress adequate reduces the band gap and is expected to sufficiently increase the photocatalytic activity of anatase TiO_2_ by inducing a red shift at the optical absorption spectrum, which has to be confirmed by experimental work and/or simulation of optical properties. Out of the cases examined, doping with Cr reduces most significantly the band gap of tensile strained anatase. Introducing N interstitial or oxygen vacancies is also beneficial. For the case of metallic doping, it is necessary to include the *U* Hubbard term correction in order to calculate more accurately the DOS. At any rate experimental work will be essential to determine the impact of temperature and other processing conditions on strain and doping. Also, the effect of deep levels has to be taken into account, as they can reduce optical activity due to the recombination of photo excited carriers. As it has been discussed in previous studies thermodynamic modelling and/or mass action analysis can provide supplementary information with DFT and experiment particularly when considering an extended range of doping and strain conditions^44–48^.

The calculations were performed using the plane wave DFT code CASTEP^49^, with the exchange and correlation interactions being modelled via the corrected density functional of Perdew, Burke and Ernzerhof (PBE)^50^ in the generalized gradient approximation (GGA), with ultrasoft pseudopotentials^51^. The kinetic energy cut-off of the plane wave basis was set to 480 eV, with a 2 × 2 × 3 Monkhorst-Pack (MP)^52^ k-point grid and 108-atomic site supercell. Calculations were under constant stress conditions. For the DOS calculations, a denser mesh of 4 × 4 × 6 k-points was employed. In our calculations the generalized-gradient approximation along with the Hubbard *U* correction was applied. The parameter U set to 8.2 eV for the Ti 3d orbitals, 4 eV for Cr 3d orbitals and 6.4 eV for Fe 3d orbitals^14,38,39^.

## Electronic supplementary material

Supplementary Information
