# Supplementary material for: A roadmap of strain in doped anatase TiO2
Source: Sci Rep. 2018 Aug 24;8:12790. doi: 10.1038/s41598-018-30747-5 (PMC6109182; doi:10.1038/s41598-018-30747-5)
Supplement: Supplementary file 1 — Supplementary Information [file 41598_2018_30747_MOESM1_ESM.docx]

**Supplementary Information**

**A roadmap of strain in doped anatase TiO_2_**

N. Kelaidis^1^, A. Kordatos^1^, S.-R. G. Christopoulos^1^, and A. Chroneos^1,2^

*^1^Faculty of Engineering, Environment and Computing, Coventry University, Priory Street, Coventry CV1 5FB, United Kingdom*

*^2^Department of Materials, Imperial College, London SW7 2AZ, United Kingdom*

Table S1. Band gap for perfect anatase TiO_2_ as a function of strain

|  | Band gap TiO_2_ (eV) | | | |  |
| --- | --- | --- | --- | --- | --- |
| Stress (GPa) | Hydrostatic | Biaxial  (001) plane | Uniaxial [001] | Biaxial  (100) | Uniaxial [100] |
| 0 | 3.15 | 3.15 | 3.15 | 3.15 | 3.15 |
| 2 | 3.15 | 3.18 | 3.12 | 3.14 |  |
| 4 | 3.13 | 3.18 | 3.09 | 3.19 | 3.19 |
| 6 | 3.14 | 3.24 | 3.06 | 3.06 |  |
| 8 | 3.07 | 3.29 | 3.05 | 3.06 |  |
| 10 | 3.08 | 3.33 | 3.02 | 3.07 | 3.28 |
| -2 | 3.14 | 3.10 | 3.17 | 3.07 |  |
| -4 | 3.14 | 3.08 | 3.20 | 3.09 | 3.15 |
| -6 | 3.10 | 3.04 | 3.21 | 3.11 |  |
| -8 | 3.03 | 2.96 | 3.21 | 3.14 |  |
| -10 | 2.96 | 2.90 | 3.20 | 3.16 | 3.12 |
